# Evaluation of a Level I trauma center provider training in patient-centered alcohol brief interventions using the Behavior Change Counseling Index rated by standardized patients

**DOI:** 10.1136/tsaco-2019-000370

**Published:** 2019-12-29

**Authors:** Doyanne Darnell, Lea Parker, Allison Engstrom, Dylan Fisher, Kaylie Diteman, Christopher Dunn

**Affiliations:** Psychiatry and Behavioral Sciences, University of Washington, Seattle, Washington, USA

## Abstract

**Background:**

Traumatic injury requiring hospitalization is common in the USA and frequently related to alcohol consumption. The American College of Surgeons requires that Level I and II verified trauma centers implement universal alcohol screening and brief intervention for injured patients. We examined whether Level I trauma center provider skill in patient-centered alcohol brief interventions improved after training and whether professional role (eg, nursing, social work) and education were associated with these skills.

**Methods:**

We present evaluation data collected as part of training in alcohol brief interventions embedded within a larger clinical trial of a collaborative care intervention targeting posttraumatic stress disorder and related comorbidities. Sixty-five providers from 25 US Level I trauma centers engaged in a 1-day workshop, with 2 hours dedicated to training in patient-centered alcohol brief interventions followed by 6 months of weekly coaching in a collaborative care model. Providers completed standardized patient role-plays prior to and 6 months after the workshop training. The standardized patient actors rated provider quality of alcohol brief interventions immediately after each role-play using the Behavior Change Counseling Index (BECCI), a pragmatic measure designed to assess the quality of behavior change counseling, an adaptation of motivational interviewing suitable for brief healthcare consultations about behavior change.

**Results:**

Seventy-two percent of providers completed both standardized patient role-play assessments. A statistically significant improvement in overall BECCI scores (*t*(41)=−2.53, p=0.02, Cohen’s *d*=−0.39) was observed among those providers with available pre–post data. Provider professional role was associated with BECCI scores at pre-training (*F*(3, 58)=11.25, p<0.01) and post-training (*F*(3, 41)=8.10, p<0.01).

**Discussion:**

Findings underscore the need for training in patient-centered alcohol brief interventions and suggest that even a modest training helps providers engage in a more patient-centered way during a role-play assessment.

**Level of evidence:**

Level V, therapeutic/care management.

## Background

Every year in the USA, 1.5–2.5 million people are severely injured and require hospital care, usually in inpatient trauma centers.^1^ An estimated 26%–63% of these injuries are alcohol-related^2^ and patients admitted with a positive blood alcohol level are at risk for suffering future alcohol-related injury.^3–5^ Brief alcohol interventions delivered at bedside in the trauma center have been shown to reduce this risk of re-injury.^6 7^ Consequently, the American College of Surgeons Committee on Trauma (ACSCOT) developed clinical guidelines in trauma centers for universal alcohol screening and brief intervention services and now requires all ACSCOT-verified Level I and II trauma centers to provide brief intervention services to injured patients.^8 9^

A 2011–2012 national survey indicated that 90% of Level I trauma centers screen for alcohol use problems and 65% reported providing an evidence-based intervention.^10^ Most trauma centers meet this requirement by utilizing existing trauma care providers such as nurses and medical social workers to deliver the counseling services.^10^ It is recommended that these brief interventions use the principles and skills of Motivational Interviewing^11^ to facilitate patient-centered conversations with patients about reducing risks or harms associated with their drinking.^12^ Previous research indicates routine trauma center providers can be trained to improve the quality and effectiveness of alcohol brief interventions consistent with Motivational Interviewing^13 14^; however, that study used a dose of training that may not be pragmatic on a routine basis and national scale.

Alcohol use problems are also only one of several behavioral health conditions commonly experienced by trauma patients; others include posttraumatic stress disorder (PTSD) and depression.^15–17^ Currently, the ACSCOT recommends that trauma centers also screen for these conditions and refer patients to treatment and encourages additional research to identify effective PTSD and depression interventions that can be feasibly initiated in the trauma center.^8^ A comprehensive and promising approach to injured patient care that can overcome common barriers to treatment^18^ and incorporates brief alcohol interventions is the Trauma Survivors Outcomes and Support (TSOS) model, which has been shown to be effective in reducing PTSD symptoms^19 20^ and the severity of patients’ concerns after injury (eg, finances, pain, psychological distress)^21^ in single-site trials. TSOS is a collaborative care intervention in which an interdisciplinary team, primarily through a care manager, supports patients early (eg, during 6 months) in their transition from inpatient to outpatient care. Support includes care coordination, delivery of brief behavioral interventions (eg, alcohol counseling), pharmacotherapy, and referral and linkage to more intensive behavioral health services, as indicated.

A multisite pragmatic hybrid implementation trial^22^ of TSOS is underway at 25 Level I trauma centers across the USA to determine if TSOS can be effectively implemented and delivered by routine trauma care providers on a national scale.^23^ The provider training protocol for the study is complete and included a brief portion of a 1-day workshop training dedicated to alcohol brief intervention delivery. The present study uses training-related data from the multisite trial to evaluate the effectiveness of training in patient-centered alcohol brief interventions among routine trauma care providers learning to implement and deliver the full TSOS model. Specifically, we observed improvement in provider skill delivering patient-centered alcohol brief interventions using standardized patient role-plays. It is known from the Motivational Interviewing literature that trainees vary in the amount and even type of training needed to become competent in patient-centered counseling, and that this may depend on trainees’ professional and training background; however, findings have been mixed.^24 25^ Therefore, we also explored whether provider professional role (eg, nursing; social work) and education level were associated with the quality of alcohol brief interventions before and after training.

## Methods

### Design

The present study is a secondary analysis using data generated as part of a 25-site clinical trial of TSOS,^23^ which uses a cluster-randomized, stepped-wedge design^26^ in which sites initiate recruitment with usual care patients and then switch on the intervention (see Zatzick *et al*, 2016 figure 3).^23^ The study biostatistician randomized sites to one of four waves using a computer-generated algorithm. The first, second, and fourth waves consisted of six sites and the third had seven. Each wave was assigned to recruit a specific proportion of usual care/control and intervention patients (eg, eight usual care and 32 intervention patients in the first wave). This study used pre–post evaluation data of TSOS provider training in alcohol brief intervention delivery generated through standardized patient role-play assessments.

### Participants and procedure

Participants included 65 trauma center providers who were primarily female (n=57, 88%) and all had, at minimum, a bachelor’s degree (majority master’s, n=38, 59%; see table 1). Social work (n=21; 32%) and nursing (n=20; 31%) were the most common professional roles. Of those reporting race/ethnicity (13 had missing data), the majority reported White/Caucasian (n=40, 77%). The 25 Level I trauma center sites selected for participation in the trial did not routinely screen and intervene to address PTSD among trauma patients. The selected trauma centers reflect the diversity found in Level I trauma centers nationally.^23^

**Table 1:** Provider demographics and comparison between those who completed vs did not complete a post-training standardized patient (SP)

Characteristics	N (%)/*M,SD*		
	Total† (n*=*65)	Pre-SP only (n=18)	Both SPs (n=47)
Gender			
Male	8 (12.3)	2 (11.1)	6 (12.8)
Female	57 (87.7)	16 (88.9)	41 (87.2)
Race/ethnicity‡			
White	40 (61.5)	9 (50.0)	31 (66.0)
Mutliracial/ethnic	7 (10.8)	1 (5.6)	6 (12.8)
Black	4 (6.2)	–	4 (8.5)
Asian	1 (1.5)	–	1 (2.1)
Age§	37.8, 10.0	36.8, 11.8	38.0, 9.8
Professional role¶			
Chemical dependency/mental health counselor	4 (6.2)	–	4 (8.5)
Psychologist/psychology trainee	9 (13.8)	–	9 (19.1)
Physician/physician trainee	5 (7.7)	1 (5.6)	4 (8.5)
Physician assistant	6 (9.2)	3 (16.7)	3 (6.4)
Nurse (RN)	8 (12.3)	1 (5.6)	7 (14.9)
Nurse practitioner	12 (18.5)	7 (38.9)	5 (10.6)
Social worker/social work trainee	21 (32.3)	6 (33.3)	15 (31.9)
Education			
Bachelors	12 (18.5)	2 (11.1)	10 (21.3)
Masters	38 (58.5)	12 (66.7)	26 (55.3)
Doctorate	14 (21.5)	3 (16.7)	11 (23.4)
Baseline BECCI**	2.1, 1.2	1.6, 1.2	2.3, 1.2

Overall BECCI score ranges from 0 (not at all) to 4 (a great extent).

*Between-group difference statistically significant at p<0.05.

†Percentages do not add up to 100% due to missing data.

‡Tested difference between White and non-White, *ns*.

§Based on n=51 due to missing data.

¶Tested difference between collapsed categories: behavioral health providers, physician/physician assistants, nurses, and social workers, *ns*.

**"Both SPs“ group based on n=45 due to missing BECCI data.

BECCI, Behavior Change Counseling Index.

All of the providers in the present study served as TSOS care managers for the trial, which included being a primary point of contact and providing care coordination and behavioral interventions to TSOS patients. At least one trauma center provider needed to be identified to serve as a TSOS care manager; however, each trauma center could determine how many and which type of other providers would contribute to the TSOS collaborative care team. TSOS care managers were identified through discussions between the site Principal Investigator (PI) and trauma center administrators and recruited by the University of Washington Coordinating Center (UW CC) to participate in a survey to collect demographic and other relevant characteristics (eg, professional role) and engage in standardized patient role-plays. Providers serving as care managers were given a link to a confidential online survey that they could complete at any time and for which they were paid US$35. Standardized patient assessments were completed within 1 month of the 1-day workshop and scheduled to occur 6 months after the workshop. Providers received US$50 per standardized patient. Additional providers could be newly identified to serve as care managers throughout the entirety of the trial; however, only providers initially recruited to attend the behavioral interventions workshop training were eligible for this study. Of the 77 eligible and recruited providers to serve as TSOS care managers, 12 declined to consent to complete the standardized patient (eight nurses, two physician/physician trainees, one social worker, and one surgeon, across seven sites; reasons were not tracked) but were able to continue their participation as part of the TSOS team. All procedures were IRB approved prior to study initiation. ClinicalTrials.gov NCT02655354.

### Provider professional role and education level

Information about providers’ professional roles and education levels were gathered through the survey and/or direct discussion with the provider. For the purposes of exploring the relation between professional role and the quality of alcohol brief interventions, we collapsed provider roles into four categories: (1) behavioral health providers (chemical dependency counselors, mental health counselors, psychologists, and psychology trainees), (2) physician/physician assistants (physicians, physician trainees, physician assistants), (3) nurses (registered nurses, advanced nurse practitioners), and (4) social workers. Highest education level was categorized into bachelor’s, master’s, or doctoral degree.

### Alcohol brief intervention training

#### Workshop training

Trauma center–based TSOS teams attended a 1-day in-person workshop just prior to the site recruiting intervention patients per the stepped-wedge design. The behavioral interventions trainer attended three in person (one for each of the first three waves) and others by audio video conferencing. The workshop covered all aspects of the TSOS collaborative care intervention and included 2 hours for patient-centered alcohol brief interventions. The trainer (DD) had co-developed and co-led multiple workshops in alcohol screening and brief intervention services and Motivational Interviewing prior to the trial with a Motivational Interviewing Network of Trainers trainer (CD). Providers’ previous training in alcohol brief interventions was not systematically assessed; however, one site incorporated TSOS into existing alcohol screening and brief intervention services.

The alcohol brief interventions were based on previous work by the research team^14^ and emphasized a patient-centered care approach,^27^ operationalized as the use of Motivational Interviewing communication skills.^11^ Providers were taught the goal of alcohol brief interventions with trauma patients is to explore potential risks associated with alcohol use and ways to reduce these risks. They were encouraged to have an empathic and collaborative style, ask open questions to elicit patient perspectives and ideas, and demonstrate understanding through reflections (ie, guesses about what a patient is thinking, feeling, or meaning to say). Providers were discouraged from confronting or warning patients or giving advice without acknowledging patient choice and autonomy. To motivate providers to use patient-centered communication skills and increase empathy for patients, an experiential activity harnessing providers’ own experiences with behavior change efforts illustrated the utility of these skills (eg, see “A Taste of Motivational Interviewing”).^28^ Discussion of case examples emphasized taking the patient’s perspective.

#### Telephone-based coaching

After the workshop training, the UW CC held 1-hour telephone/audiovisual conference calls with each site to provide support and coaching in all aspects of carrying out the TSOS model, including alcohol brief interventions. Calls were held weekly during the first 6 months of intervention delivery and less frequently (eg, monthly) thereafter. The UW CC PI and a research coordinator attended each call. The behavioral interventions trainer participated intermittently and consulted with the PI on cases as needed. Active patients were discussed during each call and a treatment plan consistent with the TSOS model was reviewed and updated. Providers were coached in the use of alcohol brief interventions if indicated, which primarily included suggestions of what to discuss with patients and reminders of how to engage patients in a patient-centered way. Provider attendance and patient-specific recommendations were documented and provided to the site team via a HIPAA-compliant electronic data capture system.

### Quality assessment of alcohol brief intervention delivery

#### Standardized patient role-plays

The quality of alcohol brief intervention delivery was assessed using standardized patient methodology.^29^ Two research staff were trained as actors to role-play a hospitalized trauma patient who was drinking alcohol at the time of their injury event (see table 2). One staff person role-played the pre-training standardized patient and a different staff person role-played the post-training standardized patient. Role-plays were conducted by telephone for a planned 20 minutes, although actual lengths varied (minutes for pre-training *M*=12.5, *SD*=6.3 and post-training *M*=15.8, *SD*=5.0). The post-training standardized patients were planned to occur 6 months (26 weeks) after the workshop training; however, the actual length varied (*M*=31.0 weeks, *SD*=7.7 weeks). Provider experiences with the standardized patient role-plays were used during the workshop training to illustrate didactic content and practice skills (eg, providers asked “what open question would you ask Angela about her alcohol use?”); however, providers were not given feedback on their standardized patient performance.

**Table 2:** Pre-training and post-training alcohol brief intervention standardized patient role-play scenario descriptions and instructions

	Pre-training	Post-training
Scenario instructions	We will be doing a brief 20-minute intervention role-play. I am going to give you a warning when we have a few minutes left for each role-play and if you feel comfortable giving a summary at that point to close out the session, you may do so.	
	I am a 21-year-old college woman named Angela. I was in a motor vehicle crash while driving home from a party. There was no blood alcohol test available, but I told the nurse I had been drinking. I have a left ankle fracture and a forehead laceration.	I am a 29-year-old computer programmer named Elizabeth. I broke my arm and collarbone. My blood alcohol level was 160 mg/dL at admission.
	You will pretend you are at bedside with me at a trauma center; your goal is to counsel me about alcohol. That is all the information about the patient I will give you to start, any other questions regarding the patient can occur during the role-play.	
Additional scenario details*	Patient engages in periodic binge episodes on weekends at parties; does not drink during the week. Patient is committed to not driving after drinking and is willing to try counting her drinks at parties and/or consider other means of socializing or relaxing that do not include alcohol.	Patient drinks nearly every weekend; averages 3–6 drinks on a night when drinking. Patient is willing to consider quitting drinking but without specific assistance; patient is not open to treatment programs or Alcoholics Anonymous.

*The standardized patient actor is trained to provide these extra details when asked relevant questions during the role-play by the provider.

#### Behavior Change Counseling Index

Immediately after each role-play, the standardized patient actors completed the Behavior Change Counseling Index (BECCI),^30^ a brief measure to assess core aspects of counseling patients about behavior change consistent with a Motivational Interviewing paradigm. The BECCI was developed to provide a brief alternative to existing measures of Motivational Interviewing that require extensive training and time to use and to be applicable specifically for use in healthcare settings. BECCI training for the standardized patient actors consisted of careful review of the BECCI manual after standardized patient actor training, which required the actors to understand the distinction between high-quality and low-quality alcohol brief intervention counseling. Eleven items are rated on a Likert-type scale with 0=Not at all, 1=Minimally, 2=To some extent, 3=A good deal, and 4=A great extent. Items were summed and divided by 11 to get an overall score ranging from 0 to 4; higher scores indicated higher quality of alcohol brief intervention counseling. Examples of items are “Practitioner invites the patient to talk about behavior change”, “Practitioner asks questions to elicit how patient thinks and feels about the topic”, “Practitioner uses empathic listening statements when patient talks about the topic”, and “Practitioner actively conveys respect for patient choice about behavior change”. The measure has demonstrated adequate inter-rater reliability and item internal consistency^30^; in the present study, Cronbach’s alpha at pre-training was 0.94 (n=62) and 0.95 (n=45) at post-training.

### Plan of analysis

#### Missing data

Thirteen providers did not complete the survey of demographic data; however, research logs captured some of this information. Eighteen (28%) care manager providers did not complete a post-training standardized patient due to no longer working at the trauma center (n=9), reported they were too busy (n=4), did not end up participating in the behavioral interventions training (n=4), and declined without a reason (n=1). Twenty-three of 25 sites had at least one provider complete both standardized patients, with a range of 1–4 and modes of 1 and 2. Three providers at pre-training and two providers at post-training had missing BECCI data due to the standardized patient actor forgetting to complete the BECCI rating form.

#### Pre–post evaluation of alcohol brief intervention quality

We observed descriptive statistics for pre-training and post-training overall BECCI scores and conducted a paired-samples *t*-test (α=0.05) to examine whether providers demonstrated improved quality of alcohol brief interventions from pre-training to post-training.

#### Association between provider professional role and education level with alcohol brief intervention quality

We explored relationships between provider professional role and education level with overall BECCI scores at pre-training and post-training using one-way between-groups analysis of variance (ANOVA); Tukey post hoc tests were used to determine which groups were statistically significantly different from each other (α=0.05).

## Results

### Provider engagement in alcohol brief intervention coaching

Although all study providers participated in the alcohol brief intervention workshop training, attendance at the UW CC–led coaching calls and receipt of patient-specific recommendations to deliver alcohol brief interventions on these calls varied across providers. On average, providers who completed a post-training standardized patient attended 11.2 supervision calls prior to their second standardized patient (*SD*=6.1) and 13 were specifically coached by the UW CC team to deliver an alcohol brief intervention with at least one TSOS patient during this time.

### Pre–post evaluation of alcohol brief intervention quality

Case-wise deletion due to missing data resulted in 42 providers for the pre–post analysis. We tested differences in demographics and baseline BECCI scores between completers and non-completers using χ^2^ tests of independence and independent-samples *t*-tests (see table 1); providers with lower pre-training skills were more likely to not complete the post-training standardized patient. Among these providers, the average overall BECCI pre-training score was 2.32 (*SD*=1.18) and 2.78 (*SD*=0.96) at post-training. Overall BECCI scores improved from pre-training to post-training (*t*(41)=−2.53, p=0.02, Cohen’s *d*=−0.39).

Given the variability in attendance at coaching calls and coaching received from the UW CC, we explored correlations between the difference from pre-training to post-training overall BECCI scores and the number of coaching calls attended as well as whether the provider was ever coached to engage a TSOS patient in alcohol brief interventions (dichotomous variable with yes=1, no=0); however, no relationships emerged.

### Association between provider professional role and education level with alcohol brief intervention quality

Provider professional role was related to overall BECCI scores at both pre-training (*F*(3, 58)=11.25, p<0.01, η^2^=0.37) and post-training (*F*(3, 41)=8.10, p<0.01, η^2^=0.37; see table 3). Behavioral health providers had higher scores than physician/physician assistants and nurses at pre-training, as did the social work group. At post-training, the behavioral health group had higher scores than all three other groups. Education level was not related to overall BECCI scores at pre-training or post-training.

**Table 3:** Descriptive statistics for overall BECCI scores at pre-training and post-training by provider professional role (n=65)

	Behavioral health			Physician/physician assistant			Nurse			Social work			Total		
	n	M	SD	n	M	SD	n	M	SD	n	M	SD	N*	M	SD
Pre-training†	11	3.23	1.00	11	1.34	1.17	19	1.41	1.04	21	2.54	0.86	62	2.10	1.22
Post-training†	13	3.67	0.40	7	2.58	0.75	11	2.16	0.76	14	2.68	1.03	45	2.82	0.95

Overall BECCI score ranges from 0 (not at all) to 4 (a great extent). Significant pre-training Tukey post hoc tests (p<0.05): behavioral health higher than physician and nurse. Social work higher than physician and nurse. Significant post-training Tukey post hoc tests (p<0.05): behavioral health higher than physician, nurse, and social work.

*Sample sizes less than 65 due to missing data: non-completed BECCI ratings (pre-training) and loss to follow-up (post-training).

†Differences between professional role categories statistically significant at p<0.05 based on between-groups ANOVA.

BECCI, Behavior Change Counseling Index.

## Discussion

Findings from this evaluation of training in alcohol brief intervention delivery indicate that routine trauma center providers, with diverse training backgrounds and professional roles, demonstrated higher quality alcohol brief intervention skills after training based on ratings given by standardized patient role-play actors. It is unlikely that the several months of coaching embedded within weekly 1-hour TSOS coaching calls contributed meaningfully to quality improvement, evidenced by lack of correlations between exposure to coaching calls and quality improvement. This is possibly due to the coaching being of low intensity (ie, primarily suggestions of what to discuss with patients and reminders to engage patients in a patient-centered way) and quantity; low quantity may be due to alcohol use problems being a common comorbidity but not a requirement of study inclusion.

The post-training scores for our providers compare favorably with other healthcare provider samples rated using the BECCI after training in Motivational Interviewing.^31 32^ For instance, a study of 61 dermatology nurses and physicians counseling standardized patients about psoriasis demonstrated a large pre–post effect size immediately after a 1-day Motivational Interviewing workshop^31^; however, their providers started out and ended up lower on the BECCI scale (pre-workshop mean=0.5, *SD*=0.5; post-workshop mean=1.3, *SD*=0.7) than did ours. Despite comparably strong scores and a medium pre–post effect size, the BECCI ratings in the present study remained in the “to some extent” range at post-training, indicating opportunity for improvement, particularly for certain types of providers. Specifically, associations between professional role and overall BECCI scores suggest that providers with training backgrounds that likely emphasize counseling skills (eg, behavioral health providers, social workers) had higher quality alcohol brief intervention counseling prior to the study training. Only the behavior health providers seem to have maintained this advantage at the post-training assessment. Trauma centers report most often utilizing nurses and social workers to deliver alcohol brief interventions^10^; however, these providers may require additional training to demonstrate skills consistent with the higher score range (ie, BECCI scores 3 or 4). Further, patient-centeredness is used in other TSOS interventions for PTSD and depression and is a value in trauma care relevant to all types of providers.^8^ If the aim is for any type of provider to be able to have behavior change conversations in a patient-centered way, our findings suggest that those without a behavioral health background in particular may need additional opportunities for training in patient-centered communication.

The question of how to pragmatically train trauma center providers to proficiently engage in patient-centered alcohol brief interventions remains in need of study. Although we observed an association between a brief workshop training with improvement in skills, research shows that feedback and coaching is needed to develop proficiency in the full set of skills believed to lead to patient behavior change.^33–35^ However, there are challenges to engaging trauma center providers in ongoing coaching,^14^ such as turnover, competing demands, and the inconvenience of scheduling with an expert coach. In addition, for some providers skills can plateau despite added supervision.^36^ Answers to how best to train providers may have to do with better tailoring to specific provider needs^25^ and appreciation that there may be some providers not well suited to alcohol brief intervention delivery.^37^ Creative approaches to make training more feasible for providers as well as efficient and effective are needed and technological advances hold great promise in this area. Web-based didactic content for alcohol brief intervention training is already widely available. Newer technologies allow providers to practice skills in computer-simulated training environments with a computerized patient and get feedback on actual performance.^38 39^ Much remains to be known, however, about how to best incorporate and use such emerging technologies with routine trauma providers delivering alcohol brief interventions or other types of behavioral interventions. Unfortunately, we also do not yet have empirical data to guide decision-making about how high scores need to be on a given quality metric of alcohol brief interventions to observe patient behavior change. Knowing what skill levels and specific skills are needed to see benefit in patient outcomes can be used to inform how much and what type of training is needed.

The provider training data for this study came from a trial in which randomization pertained to the timing of sites’ intervention patient recruitment. Consequently, the study design for assessing training outcomes was necessarily quasi-experimental. It is appropriate to say that the training was associated with improvement in skills, but we cannot rule out other possible confounds (eg, regression to the mean) or speak to causality. The observed effect sizes may overestimate what would be observed in an experimental design. A stronger design within the stepped-wedge context might include multiple pre-training and post-training assessments (eg, interrupted time-series)^40^ to account for pre-training trends in skills as well as observe the stability in post-training skills. Further, it is known that the quality of provider alcohol brief intervention skills vary in routine practice^41 42^ and additional assessments of performance with standardized patients may provide a more accurate estimate of abilities. Although standardized patients are commonly used to assess skill capacity, a caveat is that they are known to be modestly correlated with actual practice.^43^ More rigorous measurement study designs may include both samples of actual clinical care instead of or in addition to standardized patients. Given the challenges in obtaining work samples of actual clinical care,^35 44 45^ routine training of providers outside of the research context may be reliant on observation of skills practice or role-play.^46^

The assessment of provider skill was based on ratings made by the standardized patients, which has pragmatic relevance—it is increasingly popular for healthcare provider training programs to use standardized patient ratings generated immediately after a role-play to both assess trainee skill and suggest constructive feedback as a less resource-intensive alternative to paying objective raters who must listen to a recording of the role-play.^47^ In addition, standardized patients can provide the valued perspective of first-hand experience of being counseled about alcohol. One consideration, however, is that it is unknown to what extent this assessment would match an objective rating such as that by a third-party coder trained to reliably code sessions. We cannot rule out rater bias given that the standardized patients were not blind to whether the assessments were of pre-training versus post-training skills. For instance, it is possible that the post-training rating could have been uniformly more positive, unwittingly biasing results in favor of the study outcomes. The variability of scores suggests raters were using the full range of the scales at both pre-training and post-training.

There are limitations to our assessment of exposure to alcohol brief intervention coaching. The UW CC documented their recommendations for providers as to what providers should do next with patients, based on the content of the weekly UW CC coaching calls; however, detailed information about what occurred on each call and whether providers were coached in specific skills was not part of the documentation and could not be analyzed.

Findings from this study most appropriately generalize to trauma center providers within the Level I trauma care context. In addition, the sample most generalizes to providers with an undergraduate or advanced degree who self-identify as White (77%) and female (88%). The pre–post evaluation data are specific to those providers who completed a post-training standardized patient assessment. We observed a 28% drop-out, with common reasons being leaving the position and difficulty finding time to complete the assessment. In addition, providers with lower pre-training skills were more likely to drop out. It may be that the 28% reflect one or more subgroups of providers least likely to be able to engage in routine implementation of TSOS and patient-centered alcohol brief interventions as part of that service.

## Conclusions

Currently, the ACSCOT guidelines do not specify training requirements for delivering alcohol screening and brief intervention services; however, without guidelines there is a risk of variability in the quality of delivery and dilution of the public health impact of this service. In addition, our findings underscore the potential need for training in patient-centered communication skills for various types of trauma center providers who may engage patients in conversations about a variety of health behavior change topics. More research is needed to identify pragmatic, feasibly implemented training methods and the degree of skill needed for patient impact.
